# Digital health: how to govern during a never-ending data tsunami

**DOI:** 10.1038/s41746-021-00491-8

**Published:** 2021-08-10

**Authors:** David S. Muntz

**Affiliations:** 1 Baylor University, Austin, TX USA; 2 Muntz and Company, Austin, TX USA; 3 StarBridge Advisors, Boca Raton, FL USA

**Keywords:** Health care, Medical research

Few images in nature are more powerful than that of a tsunami. If only humanity were able to harness the energy associated with a tsunami. In the digital world, particularly in healthcare, a data tsunami is upon us—washing across our enterprises. To thrive, not just survive during the data tsunami—which I believe will only increase over time—data producers and data consumers need to develop a practical and pragmatic approach to govern data.

There are a great many factors contributing to this incredible yet predictable rise in data production and consumption including but not limited to a host of abbreviated items such as IoT (internet of things), IoMT (internet of medical things), SDoH (social determinants of health), AI (augmented intelligence), ML (machine learning), RPM (remote patient monitoring), “-omes” (e.g., genomes, proteomes, biomes), and the healthcare sector’s transformation to digital health. PGHD (patient-generated health data) is being collected by devices worn on our wrists, attached to or embedded in our bodies, and those in our homes. Telemedicine and telehealth interactions are extending venues where data are collected. Which data are regulated, which are relevant, which should be collected? Regardless of how others view their role—as patients or consumers—all individuals want their privacy protected, their data secured, and used in a manner consistent with their consent profile—i.e., governed.

Data are unlike physical assets in a brick-and-mortar healthcare organization. There are important differences that change the way we govern these assets. First, data assets can be accessed from any location using a wide range of devices. Like physical assets, however, they must be secured. In addition to security, there are significant privacy implications. Third, data shouldn’t be limited to possession by one person. The more data are shared, the more valuable they become. Finally, the more data assets are combined, the greater their value.

Good data governance requires principles^1^ to help guide people who produce and consume data. The three primary activities of a good data governance process are communication, coordination, and most importantly, collaboration. These 3Cs are force multipliers. Harnessing the power of a tsunami requires strong principles-based governance. Whatever governance principles you choose, it’s important to review and update them on no less than an annual basis. When businesses evolve, it’s also important to ensure your governance principles are modified to support the evolution.

Here are some ideas that can be used in the process of creating a data governance principles starter set:

**Data have value. As such, their value should be assessed, and their usage governed.** Like any asset with value, data must be protected. During the governance process, data owners and data champions should be identified. The data owner, as the term implies, possesses the data which in no way inhibits appropriate authorized sharing. As illustration, the data owner for payroll and personnel data is the Chief Human Resources Officer. For finance and general ledger data, the Chief Financial Officer has that role. There are many areas with data owners all of whom will depend upon a data champion.

The best champion should be a member of the C-suite with an enterprise view of the operational requirements of the enterprise who will ensure that throughout the data processing events, the value of the data owner’s asset is not diminished. The ideal data champion should understand how to make data meaningful and actionable.

Though generally by default the Chief Information Officer is designated as the data champion, it is increasingly common to assign a data champion in an area where analytics has evolved or will—where data consumers can get assistance in formulating the right questions, developing relevant and practical analytics to address business needs and problems. In addition to advocating for data integrity, the data champion should promote the importance of data governance using an agreed upon set of principles to help their audience understand the purpose and importance of governance. New roles are appearing: Chief Data Officers and Chief Analytics Officers, both with very distinct and emerging roles. Each of them has an interest in data governance and either could function as a data champion.

**Data retention and archiving are critical to protection of data assets***.* Create governance principles to address data retention and archiving. Some retention requirements are set by regulatory bodies. Timing impacts the value of data—over time some data improve, some become stale. Good data hygiene requires revisiting your data inventory and determining the relevance and utility of data. Those two factors and many others, particularly data consumer needs, will affect both retention and archiving.

In addition to principles, create policies and processes and utilize technologies to protect data integrity from production through consumption. Integrity^2,3^ is more than just making sure that during handoffs, data are not changed. For data to be understood, data need context. Is 98.6 a temperature or a weight? Context counts. When data are standardized and/or transformed, in the previous example from Fahrenheit to Celsius, from pounds to kilograms, the inherent meaning of the data must not be compromised. Training is essential to the data integrity process and should be required by policy. In order to ensure usage, it should be easy, accessible, and required as part of the annual security training for all users regardless of their employment relationship with an organization— that includes employees, physicians and other care providers, agents, and consultants.

**Data privacy and security are critical, and any violation of policy should be dealt with decisively and consistently.** The first act in a patient-provider relationship is the determination of consent, and appropriately so. The patient-provider relationship is based on a sacred trust. Every appropriate measure must be taken by data collectors and producers to protect that sacred trust, to ensure that the intent of consent is honored. During ideation—prior to design^4,5^ and build phases—of data-based information technologies and IT-enabled processes, attention to data privacy and security is required. Waiting until design or later is riskier, more expensive, and less effective than addressing them as primary elements in early discussions. Governance committees must make the case for including privacy and security in the earliest discussions of creating or acquiring data-based systems.

**Data must be carefully defined.** Governance requires input from both consumers and producers to ensure that meaning is consistent during processing and throughout its life cycle. Ambiguity is the enemy of common understanding. Minimization of ambiguity should be an objective. Perspectives on data, however, are often different and these different perspectives may actually increase the value of data.

**Agree upon a single source of truth.** The source could be a class of users or an information system. It’s important to identify and catalog both. Data sharing is a more complicated notion than it may seem. Too often people copy data in a system to share it without supporting processes to ensure that when the original source of truth changes that recipients of the copies are notified. Sometimes, the definition of data changes—for example, the term “admission” has changed as reimbursement rules evolved. When definitions change, old data and saved reports must be explored to understand the impact of a definitional change. The data governance committee is the body who can understand and address the implications of such a change.

According to a survey performed by KPMG and Forrester, only 34% of decision-makers say they feel confident in their data^6^. It is important to discuss the relevance of the source of truth when data are shared. Sometimes a snapshot in time is sufficient; sometimes only the original source of truth will suffice.

Changes at the source, whether user or system based, should be communicated to the data consumers.

**Patient health data are approximations of people and will be incomplete.** When I was a CIO, I was often asked by Board members, “Why can’t you share data like my bank does?” In response, I pulled a penny from my pocket and explained the difference was based on that US minted penny. Every financial activity in which a bank is involved has the incredible benefit of a common unit of measure—the penny. Every banking transaction can be described by how it impacts or how it is impacted by that common unit. Even for international transactions, there is a way to translate the value of the international currency back to the US penny.

When collecting patient data, however, there is not a standard unit of measure. We cannot truly represent patients because each of us is so unique—and that uniqueness changes during every minute of the day. We are more than our diagnoses, more than our lab results, medications, patient and family histories, and we’re more than the wide range of “-omes” (e.g., genomes and proteomes) that we’re just beginning to collect. We are more than everything that falls into the category of social determinants of health. We are more than our collection of our aspirations. The only real representation of a patient is the analog patient themselves. We must acknowledge that even the most comprehensive patient data set is just a weak representation of a real person and work within those limitations.

**Data governance exists in every organization. It must be disciplined and communicated effectively to be effective.** Even organizations with no formal data governance model are governing data—randomly and inconsistently, a relatively common situation (sometimes referred to as the anarchy architype^7^. In my experience, formalized, documented, and disciplined enterprise data governance is essential in order to achieve and maintain consistency, alignment, and compliance.

**Effective governance models are lightweight, flexible, agile, and resilient**—able to respond quickly to emerging situations as we have seen during the pandemic. Sometimes, the complexity of an organization requires the use of a federated model based on either geography or some other organizational requirement. Regardless of the model, central agreed-upon principles and proper representation from a diverse set of stakeholders are important. If you have an existing model, revisit and refresh it annually. Rapid response is the new norm. A governance process must embrace rapidity without compromising integrity, access, privacy, or security.

For research facilities whose activities involve human subjects, the Institutional Review Board (IRB) should be involved in data governance. As the FDA notes^8^, “The purpose of IRB review is to assure, both in advance and by periodic review, that appropriate steps are taken to protect the rights and welfare of humans participating as subjects in the research.” Governance is a way to monitor and ensure adequate and resilient protections are in place and evolve as research progresses.

Early in the experimental design processes, researchers should develop data capture and management schemes that protect the sacred trust referenced earlier. They should share those plans with the data governance body. If collaborative research activities require sharing patient data, the IRB should encourage, perhaps require researchers to create and submit a data sharing plan to the data governance body. Members of the IRB who are familiar with research plans and activities can be invaluable contributors to data system design activities.

**Governance requires an understanding of both data production and consumption**. Those individuals responsible for data governance should understand both data production and consumption. It’s important to have the right representatives on the data governance committee to understand both production and consumption. There are many questions that must be addressed. Why are data collected? When should they be collected? How will they be protected? How, why, when, and with whom are they shared? Are we prepared to meet the data demands for today?

More importantly, how do we prepare for the future? Having started my career in a cancer and leukemia research institute, the answer for our organization was simple. We made the decision to collect all available data because we thought we were not smart enough to distinguish between all that was important and all that was not. That approach proved fortuitous when we used data discovery tools (There are many options available. At the time, one of the tools we used was Duncan’s Multiple Range Test^9^) which helped us find statistically significant patterns in the data that our researchers had not anticipated. Those findings were rigorously reviewed, tested appropriately, and then incorporated into our protocols and approaches to treatment. Now that data storage is so cheap, this approach makes even more sense now than when it was posed 30+ years ago.

## Conclusion

The data tsunami has arrived and will continue to grow in intensity. Do not be overwhelmed by it—manage it. Done well, data governance can improve communication, coordination, and collaboration which shortens timelines and accelerates the realization of benefits. Use principled data governance to help harness the data tsunami’s power for the good of all participants in healthcare and wellness.
